# American Dental Association

**Published:** 1895-11

**Authors:** 


### Reports of Society Meetings.

### AMERICAN DENTAL ASSOCIATION.

(Continued from page 630.)

*August 7, 1895.—Second Day.—Morning Session.*

THE meeting was called to order by Dr. Watkins, the Vice-President, Dr. Crawford still being ill.

The secretary read the minutes of the previous session, which were approved.

The secretary moved that the communication read by the corresponding secretary regarding the Memorial Museum be referred to the Committee on the Horace Wells Memorial, of which Dr. Thomas is chairman. Carried.

The secretary read a communication from the Dental Society of the State of New York in regard to restraining the use of secret nostrums and preparations. The same was referred to the Section on Materia Medica. Dr. McKellops asked to have the resolution which was adopted in Boston, relative to the same matter, read before this Association.

Dr. Walker, of New York, moved a vote of thanks to the Mayor of Asbury Park for his kindness in extending the courtesy of the borough to the American Dental Association and in sending the band to furnish music, which motion was unanimously carried.

### DISCUSSION OF PAPERS READ LAST EVENING.

*Dr. Stainton.*—It is a little unfortunate that the combination of circumstances is such that papers requiring a great deal of care and preparation have to be passed without much discussion. I want to reiterate a point or two I made last night. I think some of the lessons drawn from the study I made should not be lost sight of. It is very easy to manufacture dental schools. To manufacture them of good character is another thing. We should pay some heed to that question. I have more faith in the Association of Faculties than they have in themselves. It is very easy for an outsider to have faith in a thing, whereas the people right inside of it have no faith themselves. I do not think it is too much to ask that the Faculties Association insist upon their permission being asked for the formation of dental schools. We do not say that pharmaceutical schools shall ask our permission, but only our own schools, and surely we have a right to do that. I want to call your attention to the fact early enough. The lessons taught by the figures I showed you we should not forget. People ask me if I have not over-estimated the figures, but I assure you I have not done so. It occurred to me that at this meeting we might get better data than I gave last night. I attempted to get the number of persons that constitute your practice. What I mean is, not people who come to have a tooth extracted, but persons who have had work done for them by you, teeth cleaned, a filling or two put in,—never mind if they have afterwards gone to some one else. I think it would be a valuable thing to have.

*Dr. Crouse.*—May I offer a solution of this problem? That is, not to allow the colleges to charge a fee for the clinical work. They start first with men forming themselves together to make money, and they put out the sign "Dental Infirmary" or "Dental College." So they run on until some individual comes along and buys up their charter and gets some one to lecture. Then, after a year or two, they apply to the Association, and the Association sees no way of shutting them out. In politics or anything else, when the situation gets so bad that the people cannot stand it any longer, the country is aroused and it regulates itself. I have the same faith in the college work. It may be well to look into this question from a business stand-point. It is well known that the infirmaries of colleges are a source of revenue. I have been asked to join one of these institutions, and to help them along with my journal. There are many dentists who do not belong to any society, and who do not associate with anybody. There are many men who call themselves dentists who are really not so. They move from place to place. If this Association could get the different societies to affiliate themselves with it, and if the State societies would get the good men in the State who do not belong to any society now, they could do good work.

*Dr. Jack.*—You will not have failed to observe that the purpose of the second paper of last evening was to show the necessity for care in respect to the admission of new schools. In order that a higher level might be reached, the National Association of Dental Examiners, in looking over the subject and in determining what procedures they could take that would assist in bringing out a higher level, formulated the present rules governing their action in the future. It is headed, "A Plan of Requirement for the Recognition of New Dental Schools." I might have stated to you before that we had no definite rules regulating our action. Each dental school which may in future come before the Association for recognition must have a teaching faculty composed as follows,—to wit: At least three professors of dental subjects,—namely, for operative dentistry, for dental prosthetics, and for dental pathology and therapeutics. For medical subjects there must be at least five professors,—for anatomy, physiology, chemistry, pathology, and materia medica. Its students must also be taught the subjects of chemistry and bacteriology in laboratories adapted for the purpose, and under suitable instructors. Such special school must possess, in addition to suitable lecture-rooms, a dental infirmary and rooms suitable for manual training for operative dentistry, and must furnish systematic instruction to its students. All of these provisions are to be determined by the Board of Examiners of the State within which is located the school making application to this Association to be admitted. The purpose of these rules is to restrict the recognition of new schools that are not up to the standard. The result of such action must be to commence a levelling upward of the schools which are below this standard.

*Dr. Shepard.*—I have been exceedingly pleased with the remarks of Dr. Crouse, and, in my opinion, he has presented the only solution of this question. There is not a dental college in this country but is practically telling falsehoods all the time. I have spoken on this subject before. The impression is given out, if not explicitly stated, that the fees charged in the infirmary are to cover the cost of material. I think I do not overstate it when I say that this is the universal idea which is enunciated or implied by every dental college in the country. It is a downright falsehood, whether expressed or implied. What is the cost of material in putting in oxyphosphate fillings or amalgam fillings? Is it customary to charge a nominal fee—twenty-five cents—

for an oxyphosphate filling and twenty-five cents for an amalgam filling, or is it not the general custom? A college which presents to the public the idea that the services are gratuitous, for the benefit of the public or the education of the student, and which charges as much as five cents for an oxyphosphate or an amalgam filling, overcharges from the cost of the material, and the balance goes to the profit side. I presented some figures showing the different prices charged for gold fillings at the different colleges. One college charged seventy-five cents a sheet for the gold, and this college, to make its infirmary more profitable, served out No. 3 foil instead of No. 4 because it was cheaper. Another institution charged forty cents a sheet for No. 4 foil, which was a fair and honest charge, including wastage. If there is this universal impression given out in regard to the services being free and the charge being for the cost of material, we all know whether that is true or not. If the infirmary is an element of large profit, and I am told that in some colleges the profit from the infirmaries is from ten to fifteen thousand dollars per year, the solution of Dr. Crouse is an admirable one, and will meet the case.

For some years I have been an advocate of an answer to the question why, in the minds of liberally-educated people, particularly physicians and people most familiar with eleemosynary labors, we have not had granted to us the rank we claim, either as a liberal profession or a specialty of medicine. The great reason why that has not been done is that, in the line of thought in connection with medical services, we are not a liberal profession. Where, in this country, can you point to a dental hospital, pure and simple? There is not one. Go over the list of twenty-five thousand dentists, and how many of them are giving up even half a day per week for the service of the poor? You can count them on your fingers. Look at the medical profession and the hospitals. Every town and city of twenty-five thousand inhabitants has its hospital. The services are free; the physicians go there day after day, giving more or less of their valuable time to an effort to benefit and ameliorate the condition of the sick and suffering. When we can hold up our heads and look the medical men in the face and say we are liberal, not with our money, but with what is more valuable, our skilled services, then can we claim to stand on a par with the medical practitioner as a liberal profession, doing our duty under the great, broad principle which overlies and underlies and permeates through all ages,—medical service to people to save them from and to alleviate their suffering.

I intended to write upon this subject. I advocated at the recent Congress to have a committee appointed to report on the care of the teeth of the poor. The report did not amount to much, but it is a vital question for us to take up,—What shall we do for the teeth of the poor? We must consider the question sooner or later, or we shall fail to grow to the stature which we aim for, and which some day we shall arrive at.

*Dr. Truman.*—There has not been a year since I have been connected with this Association that some one has not assailed the colleges of this country. When a man stands up here and asserts that there is not a college in this country that is not a fraud, I think it is time to stop and consider what we are doing. It is not true of all colleges. I know very well that in the college with which I am connected no such state of things exists, and I believe it to be true of a majority of the colleges. Work for the poor is done at the lowest possible cost price. Men talk about doing work for nothing. I do not know that any man here works entirely for nothing, nor do I think it is good policy as a rule to give a thing without some remuneration. Men come here and make charges against educational institutions that have been for the last forty years building up the profession, and have made it what it is to-day, and it is a disgrace to this body that they listen to it. Talk about treating the poor! I have had over thirty years' experience in this business, and I know it is impossible to get the very poor into the colleges, because they cannot spare the time to come there. Time is money to them, and when you talk of comparing dental schools with medical dispensaries it is absurd. A man can go into a dispensary and have the prescription made out for him in five minutes; in a dental college he must wait three or four hours before he can be served. There is no comparison. I am tired of this thing. I have heard it year after year. I know the colleges are doing their best to elevate the profession.

*Dr. Smith.*—The colleges *are* doing charity for the poor. The institutions with which I am most familiar have infirmaries to treat the poor. We go to the institutions and pay the bills of those who are operated on in the hospital by the week. I only refer to this to assure you that the colleges are doing charitable work for the poor, and many of the infirmaries are only self-sustaining. Dr. Shepard is mistaken. He does not know what is being done. We pay the bills of the very poor in the hospital, and we take care of the orphans gratuitously, without any charge whatever.

*Dr. Walker* (of New York).—I did not expect to speak upon this subject in any way whatever, but after listening to the three papers which were read last night and the discussions following them, I must ask, What have we, as dentists, learned? We have learned that there are some dentists connected with this Association who think we have too many dental colleges, and others who think we have about enough. It has narrowed itself down to this: that there is room for many more dental colleges if they will only place their standard higher. That is all we have gained from this discussion. It has ended in a dispute among members of different colleges, and I move that the discussion be closed.

Discussion closed.

Dr. Guilford read the report of the Section on Nomenclature, with relation to changing certain terms now in use, and calling attention to the proper pronunciation of technical terms. Dr. Molyneaux read a paper on the same subject, and was followed by Dr. A. H. Thompson, who read a paper entitled "A Basis for Dental Nomenclature," of which an abstract follows:

That dental nomenclature is in a barbarous condition is admitted by all. Like all systems of terminology, ours is more or less arbitrary and artificial, and often applied without appropriateness or discrimination. Many names have come down to us, some of which are awkward and insufficient, but must now be accepted because established by usage, and it would be impossible to change them. It is generally conceded that our nomenclature is greatly in need of correction and codification, and the first thing to be considered is a starting-point. To all students of the subject it would seem that this is furnished all ready to our hand by Dr. G. V. Black, in his admirable study and *résumé* of the subject given before the World's Columbian Dental Congress and published in the transactions. This great paper is a landmark in the history of dental nomenclature. Without wasting time as to the desirability of having an established nomenclature, we might as well proceed at once to formulate a code. This can best be done by the American Dental Association, which is the national representative body of the profession in this country. A decision emanating from this body would be held in respect by the profession, and a code of terms of nomenclature would be final. A list of the terms in general use should be submitted this year, known as "The Code of 1895," and lie over for one year, to allow time for discussion and criticism by the profession at large, and then be adopted with whatever corrections may seem desirable. A systematic dental nomenclature should be founded on the zoölogical system of names of the teeth of animals employed by naturalists as a basis. Three systems are indispensable,—

1. The gross description of teeth.
2. The detailed descriptions.
3. The minute location of areas on the surface of the teeth.

The matter is merely to be inaugurated at this meeting. The work must be continued from year to year, proceeding slowly in order to give ample time for discussion and suggestion.

DISCUSSION.

*Dr. Black.*—The importance of the question is such that it merits our closest care and attention. I will not undertake at the present time to speak specially of the words to be employed, but rather of the general subject. In order that we may succeed in adopting a nomenclature as a profession, great care must be exercised. But while the selection of words and rules for the use of words is of great importance, this will not succeed unless there has been a careful, judicious effort made to harmonize different members of the profession and different sections of the profession in the use of this nomenclature. This has been the great difficulty in medicine. Some of the specialties have, but for medicine in general there is practically no accepted nomenclature. If we are to have a nomenclature in dentistry, which would be very desirable, we must arrange ourselves into groups for the study of this subject and for the distribution of the use of this nomenclature. It should be taken up by the Association of Dental Faculties. The use of words that may be adopted in teaching by the members of these Faculties should be enforced, and their recognition of a college should depend upon that. This may sound a little harsh, but if you study the formation of nomenclature in botany and zoölogy, you will find that it is not as harsh as some of the proceedings that have been resorted to in those studies. It is not necessary to wait to complete this work, because a nomenclature in a science that is progressive is never complete. My advice to the dental profession is to organize on this subject and formulate it as far as they can, and require that in the teaching of students this be used. If you will do this, it will be but a few years before the dental profession will have a nomenclature that it can point to with pride, that will lessen by nearly one-half the time required in teaching any individual subject in dentistry. Talk about technical words being cumbersome! There is no vocation followed by any set of men that can succeed without technical words. The plasterer has his technical words, and their use is as closely followed almost as the use of technical words in botany, and we know that science, on account of the multiplicity of its terms, is most rigid. The boot-black makes a noun out of a verb, and the philologist must bow to him. He says, "Have a shine, sir?"

The time required for the dental student to accomplish certain results would be shortened and his work lessened if we did this thing that has been proposed. These are, in brief, the principal plans of getting at this work. This Association alone cannot do it, but it is properly the starting-point from which the whole should be controlled. It is necessary that it be organized throughout the ramifications in all the colleges of the country. If the colleges will do this, it will not be long before the entire profession will be united upon the subject. Of course, we must have discussions on this word or that word. It should not be done hastily. It requires years of work, and we should go into it with the view of following it up continuously. Even when we have it formed as perfectly as we can, as long as we progress we must continue the work. It has been mentioned that we should attend particularly to the nomenclature in English. Let me say that we cannot attend to this for the English language alone. Whatever we do here to-day is not only for the English language, but for the German and French. What affects these three great languages affects all others. In the selection of roots of words, particularly, we should not only look to their usage in the English language, but to the French and German as well. Take the word "abrasion," for instance. We speak of teeth being abraded when we find them worn by mastication. A French author uses that word for the operation of removing calculus from the teeth, and he has followed the root as closely as we have in English. We want to follow all the changes of meaning in these three languages particularly.

As we follow words we find that they have changed somewhat in their meaning in different languages. Some man should be found who is inclined to work in this direction, and who has the proper education in languages and taste, to take this up as a specialty and be the leader of the committees that are formed on this subject. We may find some dentist who has that peculiar tact and education as well as the inclination necessary to make him successful in following up this subject.

*Dr. Stellwagon.*—In the matter of nomenclature and the use of words in general there has been in the past a dread of the subject, caused by the fact that our dictionaries are so enormously large now that we all acknowledge that we can never conquer them. I found a little plan, which may not be original, but which has been of such great assistance to me that I adopted it and recommended it in the Philadelphia Dental College to the young men who come there. I find that it seems to have acted quite as well with them as with me. Inasmuch as those words we use are like so many implements, it is not good that we should use them in such a way that their edges become blunt or ragged. We should keep them sharp. We should use each word as it is intended to be used, and not be loose in throwing them together, as a careless dentist might throw his cutting instruments by shaking them together loosely in a drawer, which we all know would affect their edges. The most celebrated authors—men like Carlyle, for instance—were famous for their command of language. I find that they really used comparatively few words. I found that the ordinary duties of life can be explained and carried on with some two or three hundred words. This rather emboldened me to see what I could do with the words that technically belonged either to the dental profession or to the study of physiology. I boiled them down to something less than two hundred roots. I took these two hundred roots of words and gave the word with its ordinarily accepted definition. I found in that way that I could make a scaffold upon which I could hang other words. Thus, for instance, having mastered two roots of any ordinary word, we would very quickly, if we mastered two more, have a combination by which we could manufacture more. Some of the young men came to me and requested that it might be printed, and we now print these two hundred roots, and then, with a blank page opposite the printed page, note can be made of other words as fast as we desire to add to that vocabulary. The consequence is that, with a little book that takes up hardly any room, we can keep ourselves in much better touch and keep our words in much better condition. I would recommend that to those who feel the necessity for it. I recognize my imperfections, but I also recognize that I have plenty of company in that matter. When I read the Queen's English, the Dean's English, and good and bad English, I came to the conclusion that I was in good society.

*Dr. Hunt* (of Indiana).—I have heard the word which is spelt "nomenclature" pronounced nomen′clature by men upon whom I rely, and I should like to know what shall be the Bible and textbook of the philologist if not the "Century Dictionary," and they give the pronunciation as no′menclature.

*Dr. Guilford.*—I found that the accent was on the first syllable, which is "no."

*Dr. Black.*—The latest authority that has acted upon the pronunciation of that word, together with others, is a committee of the American Association for the Advancement of Science; and according to that committee the word should have accents on two syllables,—namely, no′mencla′ture, the accent being on the first and third syllables.

*Dr. Weeks* (of Minnesota).—The examples given by Dr. Guilford, and the question asked by Dr. Hunt, and the mental accusation of many of us that we frequently mispronounce words that we know how to pronounce, emphasizes the fact that in pronunciation, too, there is room for improvement. When the dental profession adopt a system of nomenclature, as has been suggested, which will be modified and added to year by year, we will be less apt to mispronounce and misapply. I apprehend that there is no one more competent to take up this matter than the American Dental Association, and I was rejoiced when this committee was appointed. I hope the committee will be continued, that this work may go on, and the Association recognize and adopt the work of an honest, earnest committee. Many suggestions have been made this morning; nearly all of them are pertinent to the question. Any gentleman who has taught the primary lessons knows how difficult it is to teach students definitely when you have no definite terms. You teach young men certain terms; at the first dental society they go to, gentlemen who stand as well in the profession as their teachers use the words in an entirely different way, and consequently the young men are muddled up. It seems we are on the right track, and I hope the Association will uphold this committee and further its work.

*Dr. Barrett.*—I approve of the report very highly. I wish the attention of the profession might be called more distinctly to solecisms that amount to absolute barbarism, such as "sixth-year molars," the "fangs" of teeth, and things of that kind which are extremely offensive in their application. It is the misuse of terms applied to certain portions of the anatomy that makes us the greatest offenders against all philological laws. While we desire to establish a definite rule of pronunciation for all our technical terms, these gross violations of all kinds of proper names should first engage our attention. I desire to call attention to these things, and I hope the committee will more pointedly mark out these wide departures.

*Dr. Thompson.*—I want to say that the committee must not expect the work to be done at once. As Dr. Black has said, it will never be completed. This committee will do the work as best it can, and then hand it on to its successors.

Dr. Marshall moved that the report be accepted and the committee continued; also, that the list presented by the committee be submitted to the profession for criticism and suggestions before final action is taken thereon.

Motion carried.

*Dr. Guilford.*—Dr. Stellwagon spoke of words and compared them with tools, which is a comparatively correct illustration. Words are really tools with which we work. If a mechanic had a nail to drive, and he drove it once with a hammer and another time with a monkey-wrench, we would not think much of him. Yet that is exactly what we have been doing,—using words once to express one thing and again to express something else. This report is made to show what we have done. We want it printed in the transactions, and we want every society to take up the subject and discuss it, and every man to constitute himself a committee of one to hunt up these words for himself and get acquainted with them. Next year, if we have decided upon the proper use of a number of words, let us have that list, and let the teachers in the colleges have those words. As fast as these are adopted let them be taken up by the editors of the dental journals, and if the editors and the teachers will use them we will soon bring about a very acceptable change. I would move that Dr. Black be appointed upon that committee in place of Dr. Stubblefield.

Motion carried, and subject passed.

Dr. Williams Donnelly then read a paper in which he earnestly urged that the Association consider the opportunity afforded by the National Medical Museum and Library to accumulate, preserve, and exhibit, at government expense, literature and museum specimens to illustrate our history, progress, and attainments, and to evidence, as we can in no other way, our claim as a liberal profession.

Dr. Crouse requested the Association to appoint a committee of five to examine the accounts and workings of the Dental Protective Association, and Drs. H. H. Smith, L. D. Shepard, H. B. Noble, L. L. Dunbar, and H. W. Morgan were appointed on such committee.

Section II. was then passed.

Motion made to devote thirty minutes at the special afternoon session for the discussion of Dr. Donnelly's paper.

After the chairman had announced the organization of the various sections, the meeting adjourned until 7.30 this evening.

